# Association of Preoperative Biliary Drainage with Postoperative Morbidity after Pancreaticoduodenectomy

**DOI:** 10.1155/2015/796893

**Published:** 2015-12-22

**Authors:** Chang Liu, Jian-Wen Lu, Zhao-Qing Du, Xue-Min Liu, Yi Lv, Xu-Feng Zhang

**Affiliations:** ^1^Department of Hepatobiliary Surgery, The First Affiliated Hospital of Xi'an Jiaotong University, Xi'an, Shaanxi 710061, China; ^2^Institute of Advanced Surgical Technology and Engineering, The First Affiliated Hospital of Xi'an Jiaotong University, Xi'an, Shaanxi 710061, China; ^3^Shaanxi Provincial Regenerative Medicine and Surgical Engineering Research Center, Xi'an, Shaanxi 710061, China

## Abstract

*Background*. The advantages or disadvantages of preoperative biliary drainage (PBD) prior to pancreaticoduodenectomy (PD) remain unclear.* Methods*. A prospectively maintained database was queried for 335 consecutive patients undergoing standard PD surgery between 2009 and 2013. Clinical data and postoperative complications of the 47 patients receiving PBD and 288 patients with early surgery were compared. A matching analysis was also performed between patients receiving or not receiving PBD (no-PBD).* Results*. The indication for PBD was severe obstructive jaundice (81%) and cholangitis (26%) at the time of PBD. 47 PBD patients had higher bilirubin level than 288 no-PBD patients preoperatively (363.2 *μ*mol/L versus 136.0 *μ*mol/L, *p* < 0.001). Although no significant difference of any complications could be observed between the two groups, positive intraoperative bile culture and wound infection seemed to be moderately increased in PBD compared to no-PBD patients (*p* = 0.084 and 0.183, resp.). In the matched-pair comparison, the incidence of wound infection was three times higher in PBD than no-PBD patients (14.9% versus 4.3%, *p* = 0.080).* Conclusions*. PBD seems to moderately increase the risk of postoperative wound and bile duct infection. Therefore, PBD should be selectively performed prior to PD.

## 1. Introduction

Though being regarded as the curative treatment of periampullary tumors and other lesions, pancreaticoduodenectomy (PD) is performed with high morbidity (~50%) and mortality (~5%). Obstructive jaundice is the most common presentation of periampullary tumors, being associated with disturbed coagulation, decreased hepatic function and the development of cholangitis, or other factors that can worsen patients outcome following PD [1, 2]. In addition to liver function, cholestasis has negative impact on cardiovascular function, leading to hypotension and impaired vascular reactivity, which predisposes to prerenal failure and acute tubular necrosis [3, 4].

Considering all these, preoperative biliary drainage (PBD) appeared just in time and has been performed by many centers. It is now clinically relevant in jaundiced patients and patients with ongoing cholangitis. What is more, PBD may also be warranted when early surgery is not feasible. Although evolved for more than 40 years, percutaneous transhepatic cholangial drainage (PTCD) and endoscopic retrograde cholangiopancreatography (ERCP) are the two most common techniques in preoperative biliary drainage. Although experimental, animal, and clinical studies have reported that biliary drainage brings favorable outcome pathophysiologically, it is not a clear cut when the clinical perioperative morbidity and mortality are considered [5–11]. Several studies found that PBD would not improve prognosis but increase postoperative morbidity (mostly infectious complications), thus raising the hospitalization and costs [12, 13]. Therefore, they concluded that PBD should be avoided for resectable tumors [12, 13]. However, PBD is still used for biliary decompression in selected patients with cholangitis or severe jaundice prior to PD in most centers. How much the increased morbidity can be attributed to preoperative jaundice management or to surgery itself remains controversial still. Therefore, the present study was to determine whether PBD was associated with increased morbidity and mortality after PD in a large series of consecutive surgeries.

## 2. Patients and Method

A prospectively maintained database was queried for patients undergoing standard PD (Whipple's procedure) surgery between January 1, 2009, and December 31, 2013, in the First Affiliated Hospital of Xi'an Jiaotong University. Totally, data was available for 335 consecutive patients. Patients undergoing PD with total pancreatectomy or pylorus-preserved PD were not included. Patients who had history of prior operative biliary or gastrointestinal bypass were also excluded from the study.

Clinical data was documented by retrospective chart review. Patient variables included age, gender, comorbidities (cardiac or pulmonary disease, diabetes mellitus), and tobacco use. Laboratory values included blood routine test values, liver function values, and tumor markers, which were documented prior to PBD or PD. Details of surgery parameters included tumor size, pancreatic/biliary duct diameter, blood loss, and pathological diagnosis. The most common PBD procedures performed in our center were ERCP with stent implantation or endoscopic nasobiliary drainage (ENBD) and PTCD. PBD was selectively performed to the patients with cholangitis and/or high level of total bilirubin, which was, however, mostly based on surgeon's experience but not any standards.

Standard PD was performed with antrectomy and child reconstruction. Digestive tract reconstruction was performed in an isolated jejunal loop. Child reconstruction was performed as standard procedure, beginning with pancreatojejunostomy (end-to-side invagination or end-to-side duct-to-mucosa), with pancreatic duct stenting when necessary. End-to-side hepaticojejunostomy was performed 10 to 20 cm distally to pancreatic anastomosis, and antecolic or retrocolic gastrojejunostomy was performed 50 cm distally to biliary anastomosis. One round silicon drainage tube was routinely placed posterior to the biliary and one posterior to the pancreatic anastomoses and connected to antireflux low pressure drainage bag. A 16 F nasogastric tube was routinely positioned in the gastric fundus.

A standardized regimen of antibiotics (second generation cephalosporin) was administered to most patients for 24 to 48 hours on a prophylactic basis, unless therapeutic application was necessary. The patients were allowed to drink some fluids after passing gas and clamp of the nasogastric tube. Once the patients complained of no abdominal distention or pain after oral intake of fluids for one day, the nasogastric tube was then removed. Then the patients started to eat soft and regular diet if they could tolerate in the following days. The abdominal drainage was checked for characters and volume postoperatively and examined for amylase if pancreatic leakage was suspected. The drainage tubes were removed if there was no evidence of any pancreatic or biliary leakage at days 5 to 7.

A pancreatic fistula was confirmed when there was an output of any measurable volume via a drain placed either intraoperatively or percutaneously at a later time, at or after postoperative day 3, with an amylase level three times that of the upper normal limit of that in serum according to the standard definition by the International Study Group on Pancreatic Fistula (ISGPF) [14]. Delayed gastric emptying (DGE) was defined as continuous drainage via the gastric tube of more than 500 mL/day over more than 5 days after surgery or recurrent vomiting in combination with swelling of the gastrojejunostomy and dilatation of the stomach at radiological contrast examination, also following the recommendation of the ISGPS [15]. Biliary leakage was confirmed either by postoperative radiological examination or when bilirubin levels where measured to be three times higher than that of serum in the drain output or in the fluid aspirated from a clinically significant intrabdominal collection [16]. Intra-abdominal infection was defined as fluid collections with positive cultures and obvious symptoms, associated with fever and high white blood cell count. A superficial surgical site infection was verified when an infection involving the skin and subcutaneous tissue of the incision occurred within 30 days from the operation, with purulent excretion or/and positive cultures with obvious symptoms.

## 3. Statistical Analysis

Comparisons of numerical data between the two groups were performed with *t*-test or Mann-Whitney *U* test. And categorical data was compared with the Chi-squared test or Fisher's exact test. Data were expressed as mean ± standard deviation (SD) or median value for numerical variables and percentages for nominal variables. The level of statistical significance was set to *p* < 0.05. Statistical analysis was carried out using SPSS 21.0 (Chicago, IL, USA).

## 4. Results

PBD was achieved totally in 47 patients (14%) by endoscopic placement of a plastic/metal stent (20/47, 42.6%) and ENBD (15/47, 31.9%) and by PTCD (12/47, 25.5%). The indication for PBD was severe obstructive jaundice (38/47, 81%) and cholangitis (12/47, 26%). As shown in Table 1, there was no difference of age, gender, comorbidities, and smoking quantities between patients with PBD and those without PBD (no-PBD). The preoperative total bilirubin was dramatically higher in PBD than no-PBD patients (*p* < 0.001), although no difference was observed in white blood cells count, hemoglobin, platelet count, alanine aminotransferase (ALT), aspartate aminotransferase (AST), albumin, and so forth. Moreover, patients in the two groups were not different in preoperative tumor markers, tumor size, diameters of biliary/pancreatic duct, intraoperative blood loss, or pathological diagnosis. Therefore, it is consistent with the reality that some clinicians would like to choose PBD when bilirubin level is extremely high. The median duration for PBD prior to surgery was 7 days (3–21 days).

Next, we examined whether there was any difference of the postoperative complications between patients with and without PBD (Table 2). Intriguingly, there was no difference between the two groups in any of the complications, for example, biliary fistula, pancreatic fistula, peritoneal infection, pulmonary infection, intraperitoneal bleeding, digestive tract bleeding, pulmonary embolism, chylous fistula, and delayed gastric emptying (Table 2). It seemed that PBD patients had higher incidence of positive intraoperative bile culture and wound infection than no-PBD patients, which, however, were not statistically different between the groups (*p* = 0.084 and 0.183, resp., Table 2). The most common bacteria cultured were* Enterococcus* (nine cases), gram-negative* Bacillus* (seven cases),* Staphylococcus* (four cases),* Streptococcus* (two cases),* Pseudomonas aeruginosa* (two cases), and* Candida albicans* (one case). Most complications occurring were of grade I or II according to the Clavien-Dindo classification of surgical complications [17]. Only seven patients needed reoperation due to uncontrollable biliary fistula (two cases), intraperitoneal bleeding (one case), digestive tract bleeding (two cases), and wound dehiscence (three cases).

We then asked the potential risk factors contributing to postoperative complications after PD. 24 variables were enrolled including patient conditions, preoperative biochemical values, primary disease, tumor status, and surgical procedures. Interestingly, only patients age and preoperative ALT and AST value were selected as risk factors associated with postoperative complications (*p* < 0.05, resp.), but not PBD or preoperative total bilirubin value (*p* > 0.05, resp., Table 3).

47 patients who had not undergone PBD were matched to the 47 patients who had undergone PBD (1 : 1). Characteristics of these patients were shown in Table 4. Groups were similar with regard to age, gender, comorbid conditions, preoperative liver function (including total bilirubin value), tumor markers, and intraoperative blood loss. The mean preoperative bilirubin was 363.2 *μ*mol/L in PBD and 324.9 *μ*mol/L in no-PBD patients (*p* = 0.09, Table 4).

The median hospital stay was 25 (7–60) days in PBD and 20 (5–45) days in no-PBD patients, which was not statistically different (*p* = 0.237, Table 5). However, PBD patients seemed to have regular food orally earlier than no-PBD patients postoperatively (*p* = 0.046, Table 5). Grossly, there were 20 PBD patients (42.6%) and 15 no-PBD patients (31.9%) developed at least one complication after surgery (*p* = 0.056). Interestingly, the incidence of wound infection was three times higher in PBD patients than no-PBD patients (14.9% versus 4.3%), although the difference was not statistically significant (*p* = 0.080).

## 5. Discussion

It still remains controversial whether to perform preoperative biliary drainage on obstructive jaundice patients with indications for pancreatoduodenectomy [6, 18]. Previous studies, either retrospective or randomized controlled trials, have drawn different conclusions. Some early studies have reported that preoperative biliary drainage could reduce the overall morbidity and mortality due to subsequent correction of the impaired liver function and general condition [11, 19]. However, later studies, including a very recent meta-analysis of randomized controlled trials, showed different results that PBD increased positive intraoperative bile culture, postoperative infectious morbidity, and sepsis-related death [20–23]. In the present study, we found positive intraoperative bile culture and wound infection seemed to be onefold higher in 47 PBD patients than 288 no-PBD patients, the difference of which was not statistically significant, although there might have been a bias, since patients with PBD were more likely to have cholangitis preoperatively. When the 47 PBD patients were matched to other 47 no-PBD patients with matched preoperative bilirubin, the incidence of wound infection was three times higher in PBD patients than no-PBD patients, although they were not statistically different (*p* = 0.080). The results might have been significant if the number of the included patients was higher.

The rate of positive intraoperative bile culture in our study was significantly lower than that published ten years ago by Jagannath et al. (6.9% versus 39.6%) [21]. Improvement of antibiotics administration and drainage techniques might contribute to decreased infection rate of biliary system. In our study, positive bile culture rate seemed to be slightly higher in patients with PBD than those without PBD (12.8% versus 5.9%, *p* = 0.084). It was understandable that most patients with PBD had severe jaundice and/or cholangitis preoperatively. Grossly, there were 20 PBD patients (42.6%) and 15 no-PBD patients (31.9%) developed at least one complication after surgery (*p* = 0.056). Therefore, in our present study, it seems true that PBD might be associated with increased infectious complication after PD. However, it should be noticed that with progression of the perioperative treatment of the patients the influence of PBD on postoperative infectious complications is now being lessened. For example, in our center, preventing contamination of wound from abdominal cavity. Bile is routinely collected for bacterial culture, especially in patients with suspicious cholangitis preoperatively. Therefore, sensitive antibiotics could be administered postoperatively.

Although improper biliary drainage might be associated with postoperative infectious complications, for example, wound infection and positive bile culture rate [13], PBD is still frequently performed in majority of patients with resectable periampullary malignancy (38~73%) in most centers [24]. However, PBD is not a routine procedure in our center, which has been performed in only 14% of the patients. The indication for PBD was mostly severe obstructive jaundice (usually bilirubin > 300 *μ*mol/L) and then cholangitis at the time of PBD. Besides complications, there are some disadvantages in both external and internal drainage. External drainage, such as PTCD, has the drawback of invasiveness and seeding risk and of dislodgement. ENBD causes patient discomfort or cosmetic problems because of the presence of the tube through nasopharynx. Internal drainage, such as stent placement, could cause cholangitis because of stent occlusion [25].

Although not recommended as a routine procedure, PBD should be performed in some selected patients. The indication might include the following: (1) treating symptomatic hyperbilirubinemia; (2) preventing sepsis due to cholangitis; (3) correcting liver and renal dysfunction secondary to obstructive jaundice; (4) improving the general condition of patients if resection should be delayed. In this current study, we followed strict criteria for patients undergoing PBD, which mainly included severe obstructive jaundice (38/47, 81%) and cholangitis (12/47, 26%). Although PBD has been more likely performed in patients with severe jaundice (with bilirubin > 300 *μ*mol/L), serum bilirubin seemed not to significantly affect postoperative complications after PD (Table 3). The reason might be that obstruction of bile system could be instantly relieved by surgery. Age and preoperative ALT and AST have been identified as the risk factor contributing to postoperative complications. It is unequivocal that aged people have poor immune system which hampers their recovery and prolongs the course of disease. Many complications could happen in the prolonged course of disease. Since ALT and AST are released from damaged hepatocytes, their increase signifies the severity of liver damage, which might be strongly correlated with postoperative recovery.

The optimal duration of preoperative biliary drainage has not been determined, since very few clinical studies have discussed this question. Animal studies suggested that at least 4–6 weeks is needed for complete recovery of the liver function after biliary decompression [26, 27]. However, whether such a long term of biliary decompression is necessary in reducing postoperative complications remains unknown. And prolonged PBD might be more likely to increase the risk of biliary infection, cholangitis, and bile duct fibrosis [24]. Therefore, we prefer subsequent surgery after correction of cholangitis or other complication by PBD (time duration, 3 to 21 days). A similar study has demonstrated that long term biliary drainage (≧2 weeks) had significantly higher biliary drainage-related complication rate than short term biliary drainage (<2 weeks) (25.9% versus 9.1%) [28]. Since longer hospital stays are expensive, PD should be performed once jaundice and cholangitis have improved.

There are also some defects in this study. Due to the retrospective nature, the small number of patients in PBD group might cause selection bias. We attempted to control for selection bias by matching for preoperative factors between the groups. Well-designed prospective randomized control trials are needed for validation of the effects of PBD, the best procedure of PBD, and the optimal duration of PBD. The results of the present study suggest that PBD seems to moderately increase the risk of postoperative bile system and wound infection.

## Figures and Tables

**Table 1:** Clinical baseline characters of the patients with and without preoperative PBD.

	PBD (*n* = 47)	No-PBD (*n* = 288)	*p* value
Mean age (years)	59 ± 2	57 ± 1	0.494
Male gender	28 (59.6%)	166 (57.6%)	0.803
Hypertension	8 (17.0%)	50 (17.4%)	0.954
Cardiovascular disease	1 (2.1%)	17 (5.9%)	0.487
Diabetes mellitus	3 (6.4%)	28 (9.8%)	0.594
Smoking pack-years (>20)	14 (30.4%)	94 (32.8%)	0.755
White blood cells (×10^9^/L)	7.2 ± 0.5	6.6 ± 0.2	0.282
Hemoglobin (g/L)	121.5 ± 2.7	119.6 ± 1.0	0.476
Platelet count (×10^9^/L)	201.3 ± 11.2	214.7 ± 5.0	0.311
Total bilirubin (*μ*mol/L)	363.2 ± 18.0	136.0 ± 8.4	**0.001**
Alanine aminotransferase (U/L)	91.1 ± 11.8	95.7 ± 4.8	0.715
Aspartate aminotransferase (U/L)	73.6 ± 8.0	81.4 ± 3.7	0.419
Albumin (g/L)	38.3 ± 0.9	40.7 ± 2.6	0.710
Prothrombin time (s)	12.9 ± 0.2	12.7 ± 0.1	0.500
Fibrinogen (g/L)	4.1 ± 0.2	4.3 ± 0.1	0.250
Carcinoembryonic antigen (ng/mL)	3.8 ± 0.4	5.7 ± 2.1	0.703
Carbohydrate antigen 125 (U/mL)	19.6 ± 3.1	31.6 ± 6.2	0.398
Carbohydrate antigen 19-9 (U/mL)	1062.5 ± 570.6	675.0 ± 203.2	0.471
Diameter of pancreatic duct (cm)	0.6 ± 0.1	0.6 ± 0.2	0.772
Tumor diameter (cm)	3.5 ± 0.2	4.3 ± 0.4	0.414
Diameter of common bile duct (cm)	1.6 ± 0.1	1.7 ± 0.1	0.460
Blood loss (mL)	463.8 ± 52.5	475.4 ± 33.5	0.893
Total expenditure (US dollars)	10158.9 ± 1152.5	8889.1 ± 280.5	0.129
Pathological diagnosis			0.881
Benign disease	2 (4.3%)	16 (5.6%)	
Malignant carcinoma	45 (95.7%)	272 (94.4%)	
Nutrient tube placement	8 (17.0%)	65 (22.6%)	0.393
Combined vessel resection	1 (2.1%)	9 (3.1%)	1.000
Hospital stay (days)	25	21	0.350

PBD, preoperative biliary drainage.

**Table 2:** Postoperative complications between the two groups.

	PBD (*n* = 47)	No-PBD (*n* = 288)	*p* value
Biliary fistula	2 (4.3%)	16 (5.6%)	1.000
Pancreatic fistula	8 (17.0%)	34 (11.8%)	0.317
Peritoneal infection	8 (17.0%)	37 (12.8%)	0.437
Intraperitoneal bleeding	1 (2.1%)	7 (2.4%)	1.000
Digestive tract bleeding	3 (6.4%)	11 (3.8%)	0.416
Pulmonary infection	3 (6.4%)	17 (5.9%)	0.750
Pulmonary embolism	0	2 (0.7%)	1.000
Chylous fistula	0	3 (1.0%)	1.000
Wound infection	7 (14.9%)	25 (8.7%)	0.183
Delayed gastric emptying	3 (6.4%)	27 (9.4%)	0.782
Positive bile culture	6 (12.8%)	17 (5.9%)	0.084
Time to oral intake (days)	6	7	0.127
Reoperation	1 (2.1%)	6 (2.1%)	1.000

**Table 3:** Potential risk factors contributing to postoperative complications after pancreaticoduodenectomy.

	With complications (*n* = 115)	Without complications (*n* = 220)	*p* value
Mean age (years)	60 ± 1	56 ± 1	**0.023**
Gender (male)	67 (58.3%)	127 (57.7%)	1.000
Preoperative biliary drainage	20 (17.4%)	27 (12.3%)	0.246
Smoking pack-years (>20)	38 (33%)	70 (32.1%)	0.902
Preoperative hypertension	20 (17.4%)	38 (17.3%)	1.000
Preoperative cardiac disease	10 (8.8%)	8 (3.6%)	0.071
Preoperative diabetes mellitus	14 (12.3%)	17 (7.7%)	0.232
White blood cells (×10^9^/L)	7.1 ± 0.3	6.5 ± 0.2	0.072
Hemoglobin (g/L)	121.6 ± 1.9	118.9 ± 1.1	0.201
Platelet count (×10^9^/L)	209.2 ± 8.5	214.7 ± 5.4	0.565
Alanine aminotransferase (U/L)	102.9 ± 5.5	80.0 ± 7.1	**0.013**
Aspartate aminotransferase (U/L)	87.2 ± 4.3	67.2 ± 5.2	**0.003**
Albumin (g/L)	37.4 ± 0.5	41.9 ± 3.4	0.330
Total bilirubin (*μ*mol/L)	167.8 ± 10.6	168.9 ± 15.7	0.953
Prothrombin time (s)	12.7 ± 0.1	12.8 ± 0.1	0.348
Fibrinogen (g/L)	4.1 ± 0.1	4.3 ± 0.1	0.171
Carcinoembryonic antigen (ng/mL)	8.8 ± 5.3	3.7 ± 0.2	0.345
Carbohydrate antigen 125 (U/mL)	22.9 ± 2.3	32.7 ± 7.5	0.386
Carbohydrate antigen 19-9 (U/mL)	486.8 ± 206.1	860.2 ± 271.3	0.360
Pathological diagnosis			0.477
Benign disease	4 (3.5%)	14 (6.4%)	
Malignant disease	111 (96.5%)	206 (93.7%)	
Tumor diameter (cm)	4.3 ± 0.6	4.1 ± 0.5	0.774
Jejuna feeding tube placement	30 (26.1%)	43 (19.5%)	0.209
Combined vessel resection	3 (2.6%)	7 (3.2%)	1.000
Blood loss (mL)	427.0 ± 34.7	497.3 ± 41.2	0.260

**Table 4:** Clinical characteristics of 94 matched patients.

	PBD (*n* = 47)	No-PBD (*n* = 47)	*p* value
Mean age (years)	59 ± 2	61 ± 1	0.363
Gender (male)	28 (59.6%)	19 (40.4%)	0.121
Smoking pack-years (>20)	10 (21.3%)	16 (34.0%)	0.249
Hypertension	8 (17.0%)	10 (21.3%)	0.794
Cardiovascular disease	1 (2.1%)	4 (8.7%)	0.160
Diabetes mellitus	3 (6.4%)	7 (15.2%)	0.169
White blood cells (×10^9^/L)	7.2 ± 0.5	6.3 ± 0.4	0.188
Hemoglobin (g/L)	121.5 ± 2.7	122.0 ± 1.7	0.873
Platelet count (×10^9^/L)	201.3 ± 11.2	195.0 ± 9.0	0.663
Total bilirubin (*μ*mol/L)	363.2 ± 18.0	324.90 ± 13.14	0.090
Alanine aminotransferase (U/L)	91.1 ± 11.8	103.8 ± 10.5	0.420
Aspartate aminotransferase (U/L)	73.6 ± 8.0	81.0 ± 7.2	0.497
Albumin (g/L)	38.3 ± 0.9	37.2 ± 0.6	0.318
Prothrombin time (s)	12.9 ± 0.2	12.5 ± 0.2	0.137
Fibrinogen (g/L)	4.1 ± 0.2	4.3 ± 0.2	0.387
Carcinoembryonic antigen (ng/mL)	3.8 ± 0.4	4.9 ± 3.1	0.086
Carbohydrate antigen 125 (U/mL)	19.6 ± 3.1	44.2 ± 20.8	0.182
Carbohydrate antigen 19-9 (U/mL)	1062.5 ± 570.6	467.0 ± 165.6	0.342
Blood loss (mL)	463.8 ± 52.5	412.8 ± 39.4	0.439
Pathological diagnosis			0.806
Benign disease	2 (4.3%)	1 (2.1%)	
Malignant carcinoma	45 (95.7%)	46 (97.9%)	
Tumor diameter (cm)	3.5 ± 0.2	3.3 ± 0.2	0.682

**Table 5:** Intraoperative characteristics and postoperative complications in matched patients.

	PBD (*n* = 47)	No-PBD (*n* = 47)	*p* value
Combined vessel resection	1 (2.1%)	1 (2.1%)	1.000
Biliary fistula	2 (4.3%)	0	0.153
Pancreatic fistula	8 (17.0%)	4 (8.5%)	0.355
Peritoneal infection	8 (17.0%)	8 (17.0%)	1.000
Intraperitoneal bleeding	1 (2.1%)	2 (4.3%)	0.557
Digestive tract bleeding	3 (6.4%)	6 (7.5%)	0.292
Pulmonary infection	3 (6.4%)	3 (6.4%)	1.000
Pulmonary embolism	0	2 (0.7%)	0.152
Chylous fistula	0	1 (2.1%)	0.315
Wound infection	7 (14.9%)	2 (4.3%)	0.080
Delayed gastric emptying	3 (6.4%)	3 (6.4%)	1.000
Positive bile culture	6 (12.8%)	7 (14.9%)	0.765
Reoperation	1 (2.1%)	1 (2.1%)	1.000
Time to oral intake (days)	6	9	0.046
Hospital stay (days)	25	20	0.237
